# Hypertrophic Osteoarthropathy Presenting as Rheumatoid Arthritis Mimicker: A Case Report

**DOI:** 10.7759/cureus.9271

**Published:** 2020-07-19

**Authors:** Firdevs Ulutaş, Meral Ulu, Uğur Karasu, Veli Çobankara

**Affiliations:** 1 Rheumatology, Pamukkale University, Denizli, TUR; 2 Internal Medicine, Pamukkale University, Denizli, TUR

**Keywords:** hypertrophic osteoarthropathy, elderly onset rheumatoid arthritis

## Abstract

Paraneoplastic rheumatologic syndromes are defined as clinical conditions that mimic primary rheumatic disease in the course of cancer; they generally improve with the effective treatment of underlying malignancy. Hypertrophic osteoarthropathy (HOA) is one of the paraneoplastic syndromes, and it is characterized by the combined presence of periostosis, digital clubbing, and swelling of soft tissues, skin, and joints in the distal extremities. HOA is commonly associated with intrathoracic malignancies (primary lung tumors or metastases). In this report, we discuss a patient with HOA secondary to lung adenocarcinoma, who was admitted with symmetric polyarthritis presenting as elderly onset rheumatoid arthritis. He was successfully treated with chemotherapy ablation for underlying malignancy. Anti-inflammatory drugs were also administered. HOA should be kept in mind in the differential diagnosis of inflammatory arthritis.

## Introduction

In elderly patients, the differential diagnosis of inflammatory polyarthritis generally includes many clinical conditions such as polyarticular gout, pseudogout, elderly onset rheumatoid arthritis, osteoarthritis, and paraneoplastic syndromes. Paraneoplastic rheumatologic syndromes are defined as clinical conditions that mimic primary rheumatic disease in the course of cancer; these conditions generally improve with the treatment of underlying malignancy [1]. Hypertrophic osteoarthropathy (HOA) is one of the paraneoplastic syndromes, and it is characterized by the combined presence of periostosis, digital clubbing, and swelling of soft tissues, skin, and joints in the distal extremities. HOA is commonly associated with intrathoracic malignancies (primary lung tumors or metastases), especially with non-small cell lung cancer with poor prognosis [2].

## Case presentation

**Figure 1:**
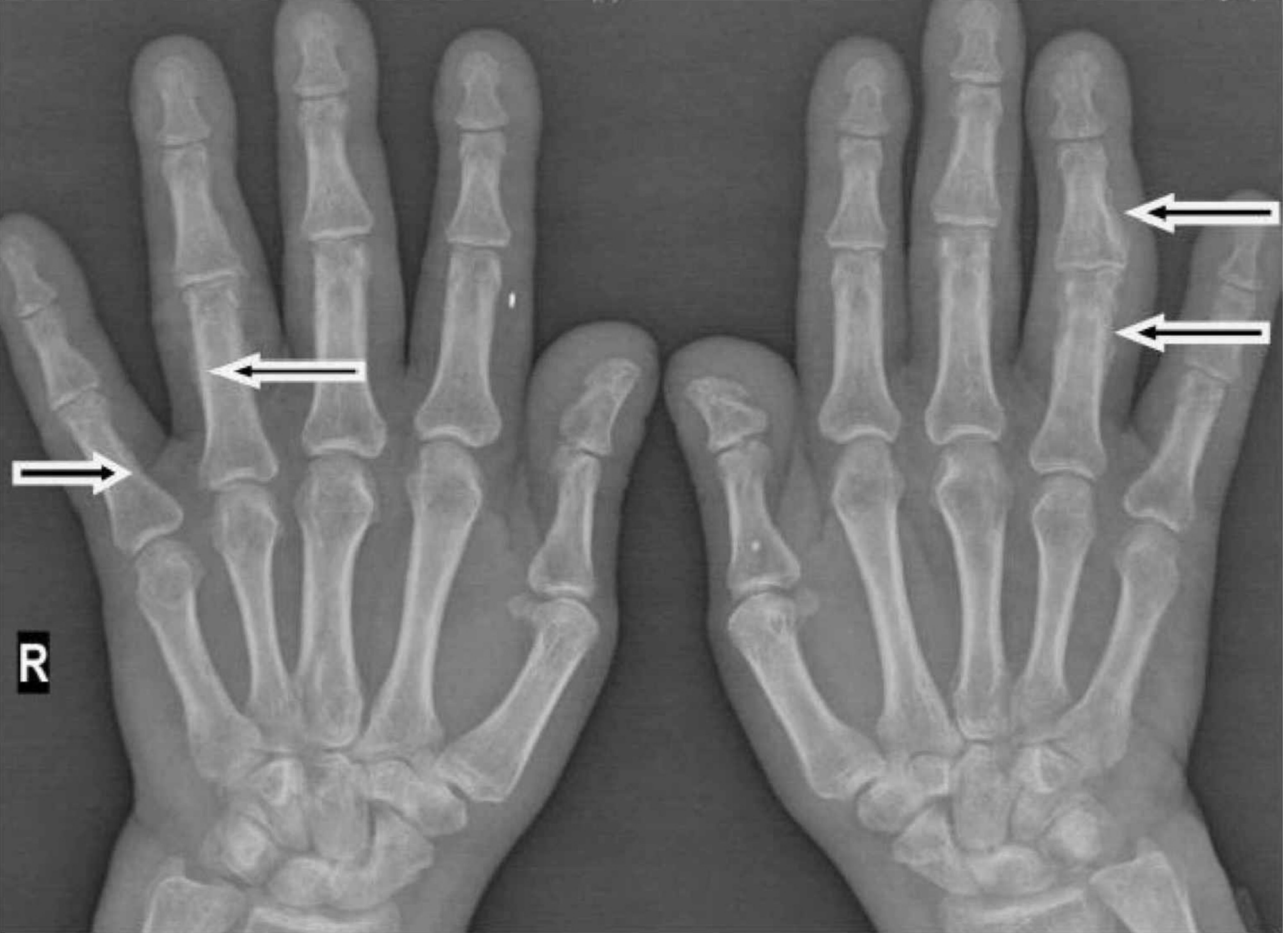
Conventional radiography image 1 The image shows periosteal reaction including proliferation, irregularity, and elevation of periosteum on phalanges (arrows)

**Figure 2:**
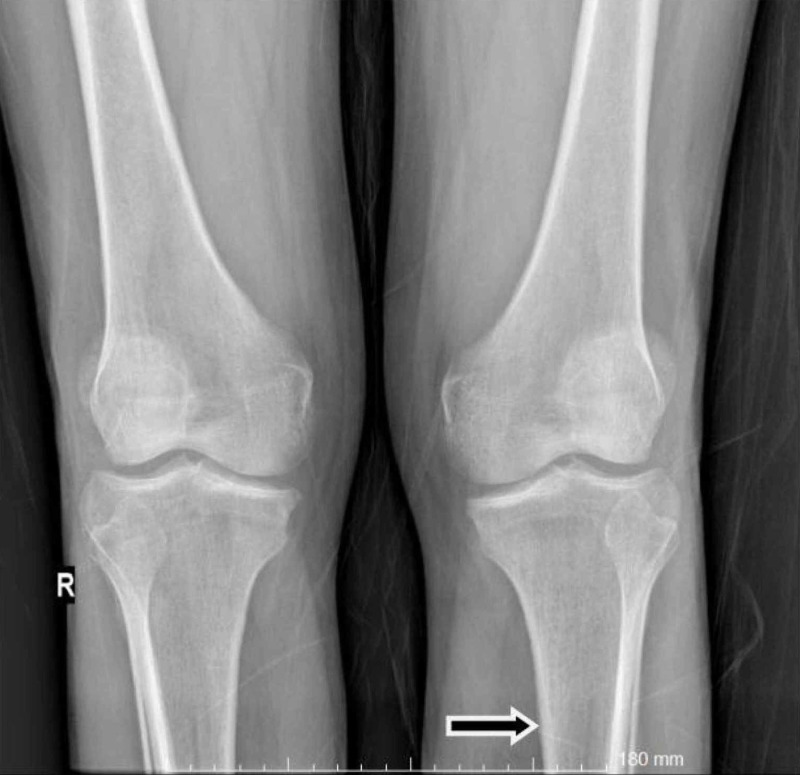
Conventional radiography image 2 The image shows periosteal reaction including proliferation, irregularity, and elevation of periosteum on the tibia (arrow)

**Figure 3:**
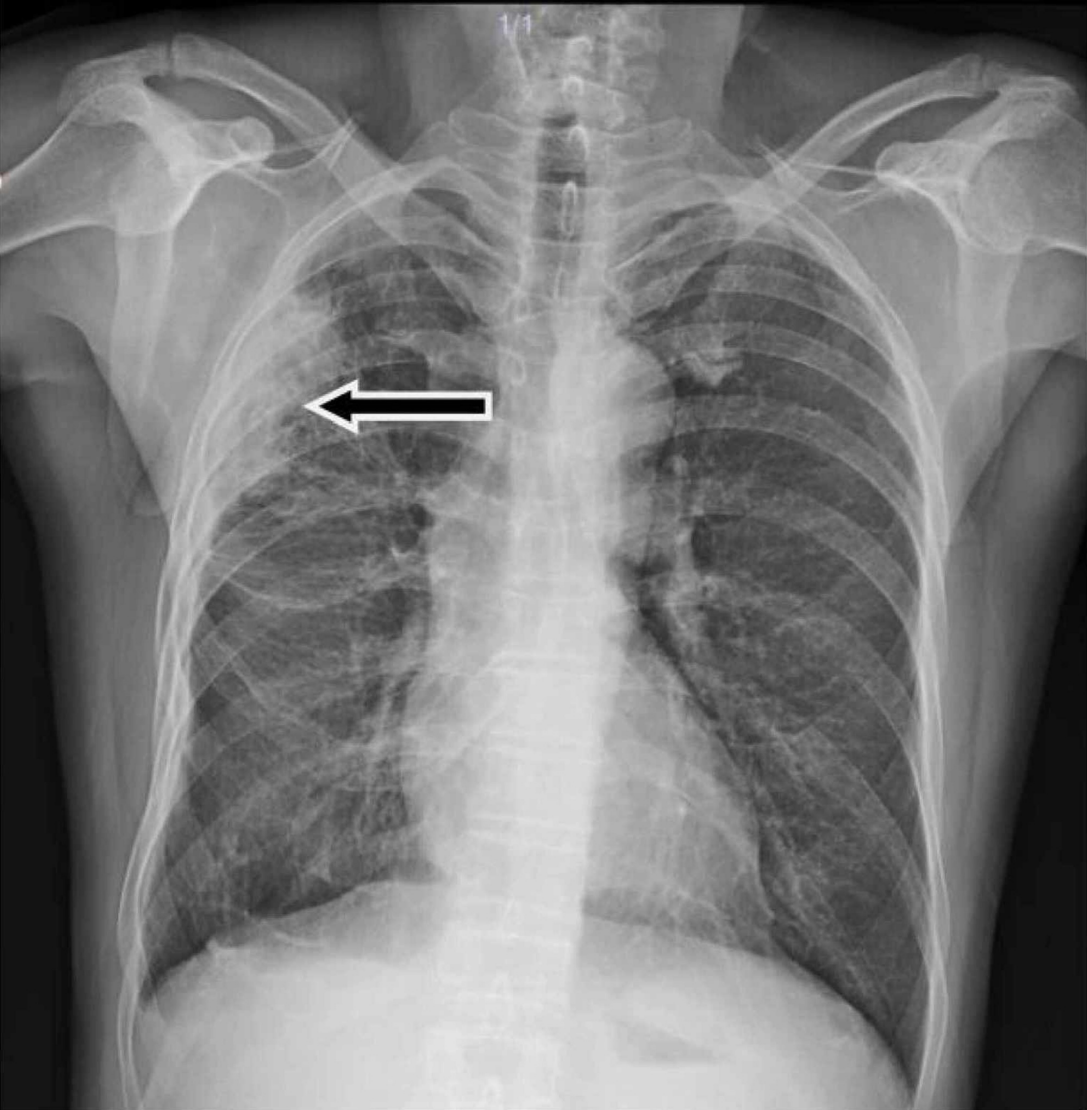
Posteroanterior chest radiography The image shows a well-defined nodular mass in the right upper lung lobe (arrow)

**Figure 4:**
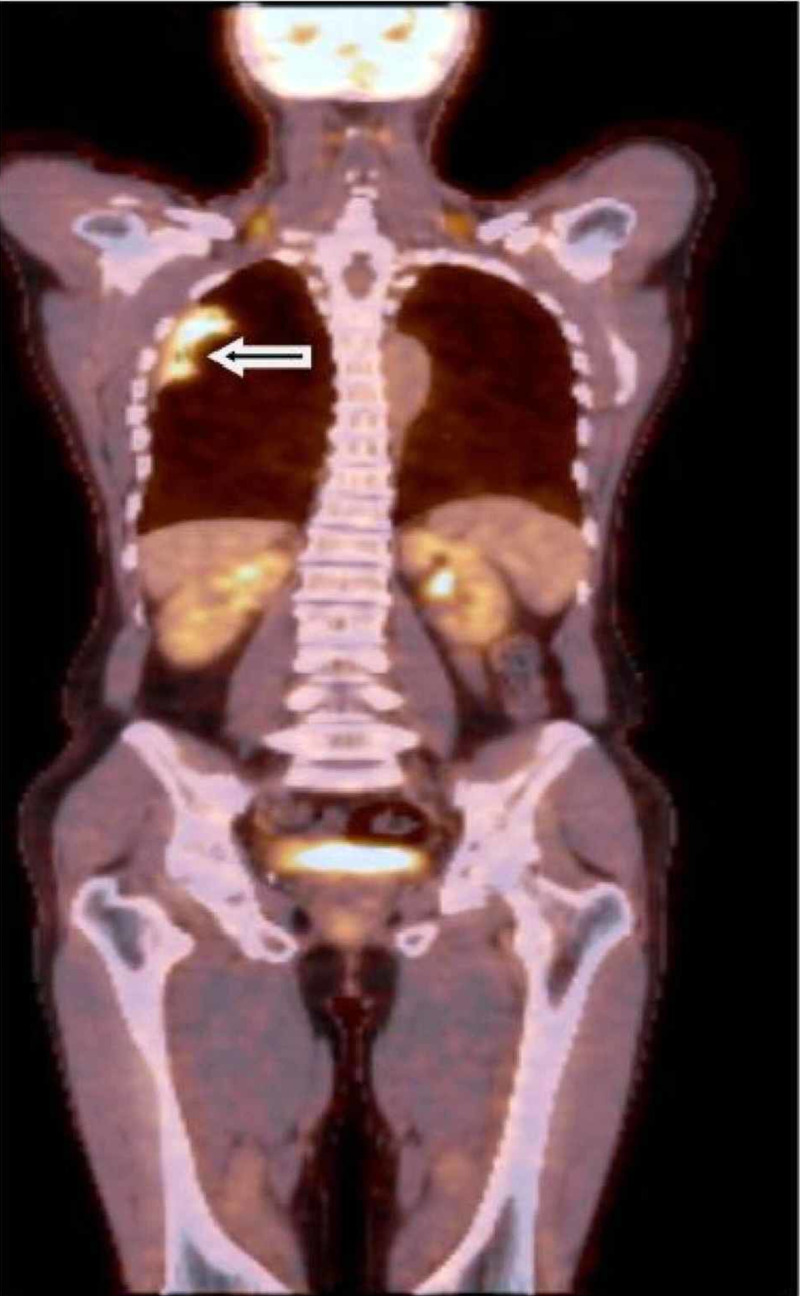
Fluorodeoxyglucose-positron emission tomography imaging The image shows increased uptake of FDG in the nodular lesion (arrow)

**Figure 5:**
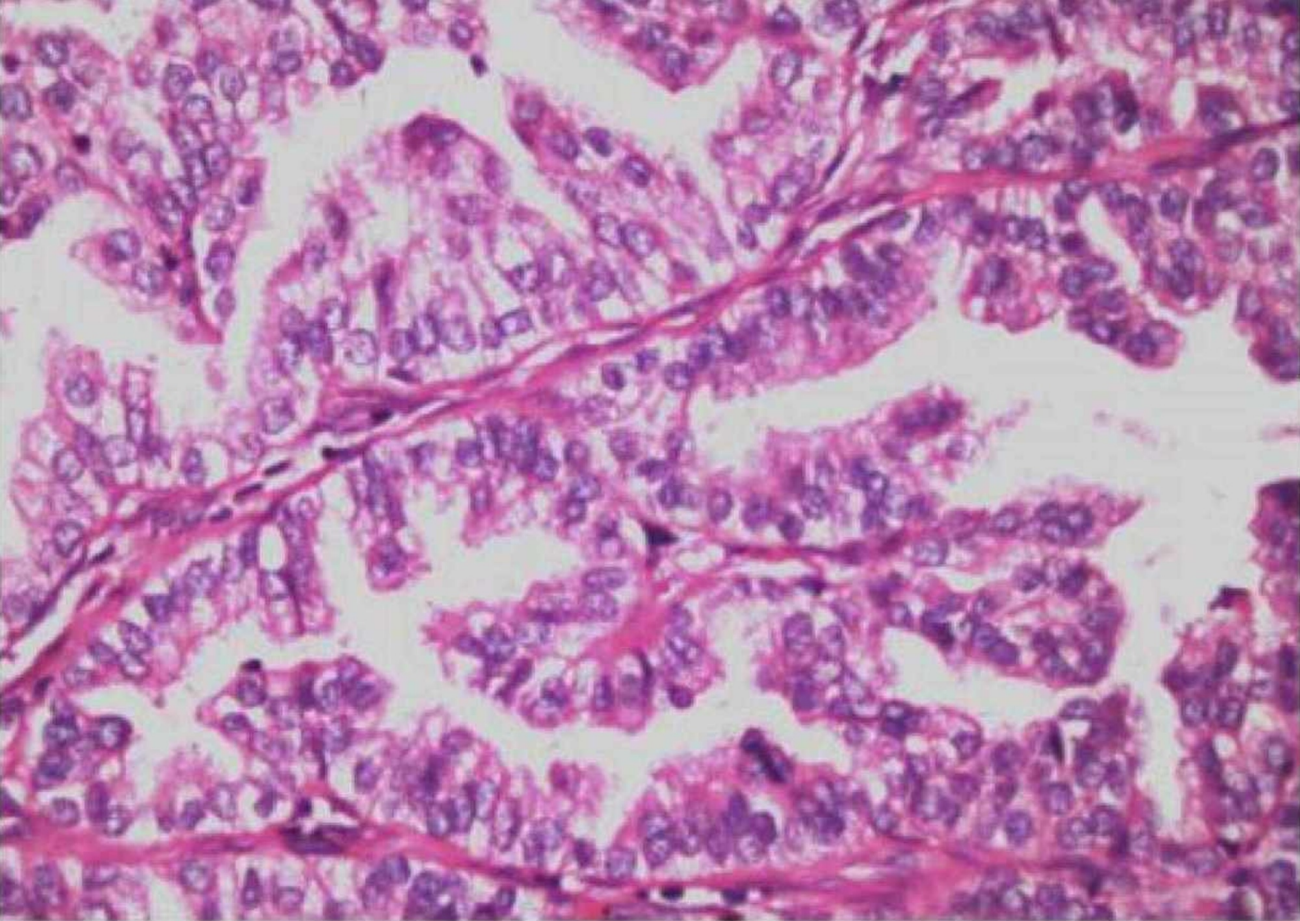
Histopathological findings The image shows lung adenocarcinoma, a round array of tumor cells with peripheral nuclear polarization (HE *200 Pathology Archive)

A 61-year-old man presented with a three-month history of worsening diffuse join pain, three hours of morning stiffness, and swelling of both hands, wrists, and knees. He was initially evaluated at the rheumatology outpatient clinic. He showed no pulmonary symptoms including chest pain, hemoptysis, cough, or shortness of breath. He had no systemic symptoms such as fever and weight loss. He had been a smoker for 10 years until he quit. On physical examination, subtle digital clubbing, symmetric arthritis of wrists, ankles, and metacarpophalangeal joints were determined. Tenderness on palpation was present on the bilateral tibia shaft. Laboratory results revealed no positive rheumatologic markers including rheumatoid factor, anti-citrullinated protein antibodies (anti-CCP), and anti-nuclear antibodies (ANA). Routine tests were normal except for markedly increased c-reactive protein and erythrocyte sedimentation rate. The conventional radiography images revealed periosteal reaction including proliferation, irregularity, and elevation of periosteum on phalanges and tibia (Figures 1, 2), and showed a well-defined nodular mass in the right upper lung lobe (Figure 3). The patient underwent fluorodeoxyglucose-positron emission tomography imaging (FDG-PET) and transbronchial biopsy, respectively. PET showed increased uptake of FDG in the nodular lesion (Figure 4). Ultimately, histopathological findings including tumor cells with peripheral nuclear polarization suggested the diagnosis of lung adenocarcinoma (Figure 5). He was treated with palliative chemotherapy. Analgesics and non-steroidal anti-inflammatory drugs (NSAIDs) were used to relieve joint pain. After the second month of treatment, the daily consumption of NSAIDs decreased and the patient's condition improved. Acute phase reactants returned to normal ranges.

## Discussion

HOA is often a paraneoplastic phenomenon as mentioned above [3]. Paraneoplastic rheumatologic syndromes may precede the diagnosis of malignancy such as the one found in our patient or may occur simultaneously. A rapid, atypical onset or progressive course of the disease, or poor response to treatment should raise suspicion among clinicians regarding the paraneoplastic nature of rheumatic diseases [4].

Vascular endothelial growth factor (VEGF), platelet-derived growth factor, and increased levels of prostaglandin E2 are well-known factors in the pathogenesis of the disease [5]. VEGF is the most known factor, and it plays a key role. High serum and plasma concentrations of VEGF in patients with lung cancer-related HOA have been reported [6]. VEGF is a hypoxia-induced agent that is produced by malignant tumors for the growth of cancer cells. Localized activation of platelets and endothelial cells occurs via the subsequent release of these growth factors. This activation stimulates angiogenesis, endothelial hyperplasia, the proliferation of mesenchymal connective tissue, and the deposit of collagen fibers [7].

Ulusakarya et al. have reported the case of a patient diagnosed with HOA and metastatic lung disease of nasopharyngeal carcinoma. Following the chemotherapy courses, the patient’s analgesic consumption decreased and HOA disappeared with complete antitumor response [8]. Management of the condition and survival are dependent on the underlying disease. Its effective treatment may rapidly lead to the resolution of symptoms.

HOA may have variable clinical presentations, mimicking various rheumatic diseases such as rheumatoid arthritis, as found in our case. Bozzao et al. have recently reported a case with HOA and lung adenocarcinoma that presented with initial symptoms including fever, diarrhea, and oligoarthritis [9]. In our case, a worsened digital clubbing prompted us to investigate further. Although clinicians are capable of recognizing advanced cases with clubbing, subtle presentations can be easily missed. Currently, there is no gold standard assay for the detection of clubbing. Increased phalangeal depth ratio or the Schamroth window test may help clinicians before clubbing is markedly developed [10]. For the subtle disease, assuming the presence of clubbing and searching for underlying malignancy may be the best approach for early diagnosis of occult cancer.

## Conclusions

HOA should be kept in mind in the differential diagnosis of inflammatory arthritis. Red flags like digital clubbing and a history of smoking among patients should raise suspicion among clinicians regarding a paraneoplastic condition in the routine clinical practice.
